# A novel synbiotic (SCM06) for anxiety and sensory hyperresponsiveness in children with autism spectrum disorder: an open-label pilot study

**DOI:** 10.1038/s41522-025-00902-8

**Published:** 2026-01-14

**Authors:** Oscar W. H. Wong, Zhilu Xu, Sandra S. M. Chan, Flora Y. M. Mo, Caroline K. S. Shea, Qi Su, Monica Y. T. Wan, Chun Pan Cheung, Jessica Y. L. Ching, Whitney Tang, Hein M. Tun, Francis K. L. Chan, Siew C. Ng

**Affiliations:** 1 Department of Psychiatry, The Chinese University of Hong Kong, Hong Kong SAR, China; 2 The D. H. Chen Foundation Hub of Advanced Technology for Child Health (HATCH), The Chinese University of Hong Kong, Hong Kong SAR, China; 3 Microbiota I-Center (MagIC), Hong Kong SAR, China; 4 Department of Medicine and Therapeutics, The Chinese University of Hong Kong, Hong Kong SAR, China; 5 Department of Psychiatry, Alice Ho Miu Ling Nethersole Hospital, Hong Kong SAR, China; 6 The Jockey Club School of Public Health and Primary Care, The Chinese University of Hong Kong, Hong Kong SAR, China; 7 Centre for Gut Microbiota Research, The Chinese University of Hong Kong, Hong Kong SAR, China; 8 Li Ka Shing Institute of Health Sciences, State Key Laboratory of Digestive Disease, Institute of Digestive Disease, The Chinese University of Hong Kong, Hong Kong SAR, China; 9 New Cornerstone Science Laboratory, The Chinese University of Hong Kong, Hong Kong, China

**Keywords:** Microbiology, Health care, Microbiota

## Abstract

Anxiety and sensory hyperresponsiveness are common in children with autism spectrum disorder (ASD), but effective treatments are lacking. Targeting the microbiota-gut-brain axis is a promising strategy. This open-label pilot study evaluated SCM06, a novel synbiotic designed to target anxiety and sensory hyperresponsiveness, in 30 children with ASD (mean age 8.2 years, 22 males). We assessed symptom improvement, compliance, and safety, and collected stool samples for metagenomics and metabolomic analysis over 12 weeks. SCM06 was safe and well-tolerated, and significant improvements were observed in anxiety, sensory hyperresponsiveness, and abdominal pain. Following SCM06 treatment, increase in *Bifidobacterium pseudocatenulatum* was associated with improved functional abdominal pain (p = 0.0011, p__adj_ = 0.054), while the abundances of valeric acid and butyric acid increased (p__adj_ = 0.004 and p__adj_ = 0.072). Key microbial species, *Coprococcus comes* and *Veillonella dispar*, were candidate mediators of symptom improvements. Further randomised controlled trials are warranted to confirm its clinical efficacy.

## Introduction

Although autism spectrum disorder (ASD) is a neurodevelopmental condition characterized by social communication deficits and restricted, repetitive behaviours (RRBs), the functional impairment experienced by individuals with ASD arises not only from these core features but also from highly prevalent co-occurring psychiatric and physical conditions. Among these, anxiety emerges from toddlerhood and affects up to 35% of young children with ASD, significantly impacting their well-being and developmental trajectories^1^. Untreated anxiety can disrupt social and academic development and increase the risk of psychiatric complications, such as depression, conduct problems, and suicidality in later life^2,3^. Despite its clinical importance, the complex pathophysiology of anxiety in ASD remains poorly understood, and no specific biological treatment is currently available^4^.

Sensory hyperresponsiveness, a core feature of ASD, is consistently associated with anxiety in young children with ASD^1^. Longitudinal studies have observed that sensory hyperresponsiveness precedes and predicts anxiety, implicating its role in the pathogenesis of anxiety^5^. Emerging evidence indicates that the gut microbiome produces metabolites relevant to brain signal transmission and inflammation to drive sensory dysregulations in ASD^6,7^. For example, the gut microbiome is known to influence brain GABA receptor expression^8^, which are relevant to the excitatory/inhibitory imbalance in ASD that underpins sensory hyperresponsiveness and anxiety^9^. Pro-inflammatory cytokines released during pathological gut-brain interactions may further amplify these symptoms^10^. Hence, modulation of the gut microbiome holds promise as a targeted treatment for anxiety in ASD through regulating sensory processing.

Among different gut microbiota modulation therapies, probiotics are appealing due to their ease of administration and favourable safety profile. A recent meta-analysis suggested potential benefits of probiotics in ASD^12^, but the results were inconclusive and lack generalizability. Most existing clinical trials of gut microbiota modulation have used overall ASD symptom severity as the primary outcome^13^. However, the recent *Lancet* Commission on the future of care and clinical research in autism emphasizes that interventions need to address the diverse symptomatology and focus on the specific profile of needs in children with ASD^14^. In other words, a one-size-fits-all approach of intervention may not be adequate to address the clinical heterogeneity of ASD, and selecting participants with shared clinical characteristics and needs in trials may yield more conclusive results^15^. To address these gaps, our research team developed SCM06, a novel multi-strain synbiotic formula (US provisional patent application no: 63/667,805), designed specifically for anxiety and sensory hyperresponsiveness in children with ASD. SCM06 combines two prebiotics (maltodextrin and galactooligosaccharide) and 5 billion colony-forming units (CFU) of four probiotic species, including *Bifidobacterium bifidum, Bifidobacterium longum, Lactobacillus plantarum*, and *Streptococcus thermophilus*. These probiotic species were selected for their ability to enhance GABA production in laboratory settings and potential in regulating the immune system^16–19^, addressing the postulated pathways within the microbiota-gut-brain axis that underpin the targeted symptoms. Here, we report the results of a pilot study evaluating SCM06 in children with ASD with anxiety and sensory hyperresponsiveness, and we also assessed changes in gut metagenomic and metabolomic profiles after the intervention.

## Results

### Subject recruitment

**Fig. 1:**
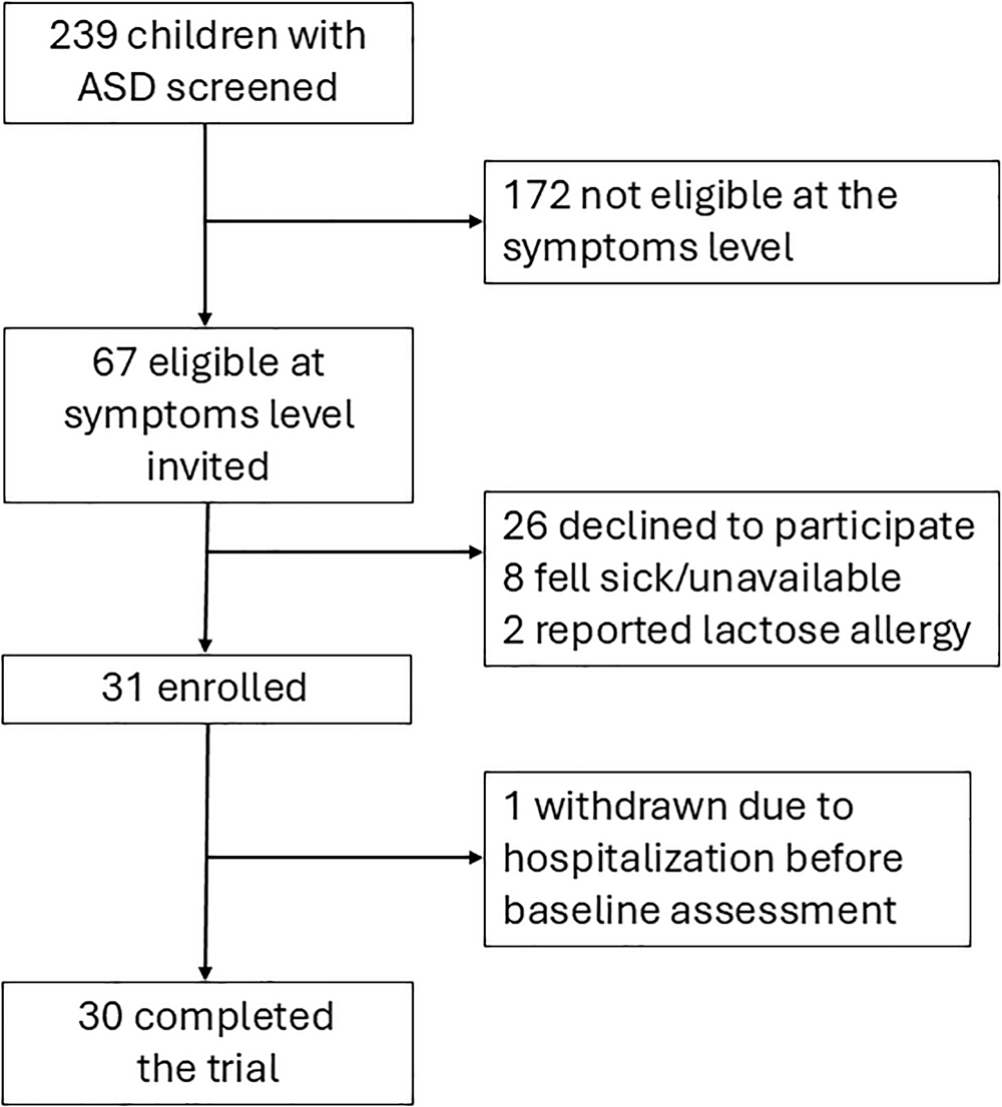
Study flow chart.

The study was conducted from December 2023 to July 2024. During the recruitment period, a total of 239 children attended the annual follow-up visit of the longitudinal study on ASD and gut microbiome. Among these, 67 (28.0%) were potentially eligible based on their ASC-ASD and SEQ scores and were approached for recruitment. There were 26 parents (38.8%) who declined to participate, and 41 expressed initial interest after discussing the study details. However, eight participants were unable to join ultimately because the child fell ill or due to scheduling conflicts. Two children were excluded because of a subsequent reported history of lactose allergy. One participant, whose parents provided written consent, withdrew before completing the baseline assessment because the child was hospitalized for a physical illness. Eventually, 30 participants (22 males and 8 females) enrolled and completed the study (Fig. 1).

### Characteristics of the study population

The age ranged from 4 to 11 years (mean = 8.2 years, SD = 2.04), of whom 17 (56.67%) had co-occurring ADHD, with nine (30%) taking stimulants, one (3.33%) taking antipsychotics, and four (13.33%) taking both. All medications were initiated and stabilized before the trial, with no changes in dosage and type during the study. No participants received new training or behavioural interventions during the study period. While there was no significant change in the estimated intake of macronutrients (carbohydrates, protein and fat) and daily total energy during the 12-week course of SCM06 (Table 1), some of the participants had dietary problems (Supplementary Table 1). These include intake of >125% estimated energy requirement (EER) (23.3%), <75% ERR (3.3%), carbohydrate insufficiency (13.3%), protein insufficiency (6.7%), fat overconsumption (13.3%) and underconsumption (3.3%).

**Table 1:** Changes in physical measurements, clinical symptoms of ASD, prevalence of functional gastrointestinal disorders over the 12-week course of SCM06

	Baseline		Week 6		Week 12		Statistics			
	Mean/*n*	SD/%	Mean/*n*	SD/%	Mean/*n*	SD/%	F/Q	df	*p*	ηp2
*n*	30		-		-		-			
Age	8.2	2.04	-		-					
Sex										
Male	22	73.33	-		-					
Female	8	26.67	-		-					
Co-occurring ADHD	17	56.67	-		-					
Usage of Psychiatric Medications	14	46.67	-		-					
Body Mass Index	16.90	3.32	16.70	2.60	16.30	2.88	1.51	2, 58	0.229	0.05
Dietary intake										
Total energy intake (kcal)	1945.64	659.85	2010.32	504.16	1928.89	648.74	0.397	2,50	0.627	0.016
Carbohydrates (g)	271.61	81.34	267.87	64.78	253.30	90.34	1.33	2,50	0.332	0.043
Protein (g)	80.50	28.26	89.08	26.84	94.72	29.86	1.22	2,50	0.297	0.047
Fat (g)	59.69	32.46	64.72	23.87	59.65	26.53	0.648	2,50	0.486	0.025
SRS-2										
Social Communication and Interaction	89.3	16.4	86.9	15.9	84.6	16.5	2.47	2, 56	0.094	0.081
Restricted and Repetitive Behaviour	20	6.09	19.1	5.12	19.4	6.15	0.35	2, 56	0.703	0.013
ASC-ASD Total score	36	10.9	30.6	12.6	30.8	11	7.83	2, 56	0.001^**^	0.219
SEQ										
Hyperresponsiveness Subscale	3.21	0.516	3.08	0.637	2.98	0.585	3.85	2, 56	0.027^*^	0.121
Hyporesponsiveness Subscale	2.51	0.629	2.38	0.63	2.36	0.721	1.09	2, 56	0.343	0.037
CBCL										
Attention Problem Subscale	70.9	8.62	69.4	9.88	68	8.83	1.99	2, 56	0.146	0.066
Externalizing Behaviour Subscale	64.3	9.33	62.6	8.68	62.5	8.77	1.67	2, 56	0.198	0.056
Internalizing Behaviour Subscale	62.6	8.74	60.8	8.12	61.4	8.68	0.84	2, 56	0.439	0.029
FGID										
FNVD	3	10.00	1	3.33	2	6.67	3	2	0.236	-
FAPD	8	26.67	4	13.33	3	10.00	8.4	2	0.045^*^	
FDD	9	30.00	5	16.67	6	20.00	2.89	2	0.236	

*p < 0.05, **p < 0.01. Linear mixed models for clinical symptom changes were adjusted for baseline scores (centered), baseline-by-time interaction (for regression to the mean), age, sex, BMI, ADHD status, medication use, and average total energy intake, with random intercepts for subjects. Cochran’s Q tests for changes in FGID prevalence (FAPD, FNVD, FDD) applied false-discovery rate adjustment for multiplicity.

*ASD* Autism Spectrum Disorder, *BMI* Body Mass Index, *ADHD* Attention Deficit and Hyperactivity Disorder, *SRS-2* Social Responsiveness Scale 2nd Edition, *ASC-ASD* Anxiety Scale for Children-ASD, *SEQ* Sensory Experiences Questionnaire, *CBCL* Child Behaviour Checklist, *FGID* Functional Gastrointestinal Disorder, *FNVD* Functional Nausea and Vomiting Disorders, *FAPD* Functional Abdominal Pain Disorders, *FDD* Functional Defecation Disorders.

### Compliance, safety, and tolerability

The participants had a median compliance rate of 95.2% (Interquartile Range = 9.52; Minimum: 52.38%; Maximum 100%), with two participants having compliance below 80%. The 12-week SCM06 course was generally safe and well-tolerated, with only two (6.67%) participants reporting self-limiting loose stools and one (3.33%) reporting reduced appetite as GI side effects. These GI side effects were mild, requiring no medical intervention. One parent reported mild worsening of the child’s pre-existing eczema during the first 6 weeks of treatment. No adverse events were reported. The two participants with low compliance did not report any side effects.

### Clinical improvement

**Fig. 2:**
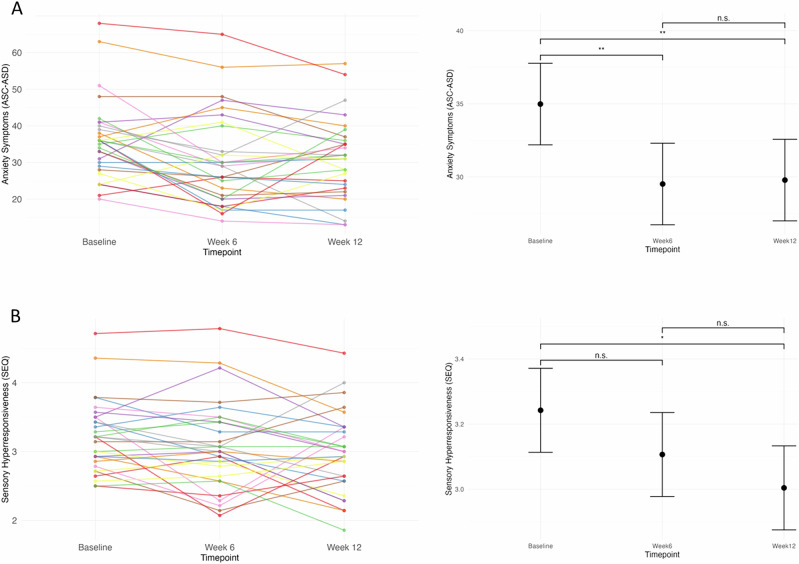
Exploratory mediation analysis of the role of gut microbial species, pathways, and metabolites in clinical symptom improvement. Spaghetti plots and overall changes in **A** Anxiety and **B** Sensory Hyperresponsiveness over the 12-week course of SCM06. *Note*. *p < 0.05, **p < 0.01. Error bars represent 95% confidence interval. ASC-ASD Anxiety Scale for Children-ASD, SEQ Sensory Experiences Questionnaire.

Using Linear mixed models (LMMs), we found significant improvement in anxiety (F(2, 56) = 7.83, *p* = 0.001, η_p_^2^ = 0.219) and sensory hyperresponsiveness (F(2, 56) = 3.85, *p* = 0.027, η_p_^2^ = 0.121) in children with ASD after 12 weeks of SCM06 (Fig. 2). The baseline-by-time interaction for assessing regression to the mean (RTM) was marginally significant for anxiety (*p* = 0.099) but not for sensory hyperresponsiveness (*p* = 0.435), indicating minimal RTM influence. The prevalence of functional abdominal pain disorders also decreased from 26.7% to 10.0% (Q = 8.4, df = 2, p__adj_ = 0.045). Core ASD symptoms and other co-occurring symptoms remained unchanged over the 12 weeks (Table 1). Post-hoc comparisons showed that anxiety symptoms decreased significantly by Week 6 (Baseline vs Week 6: *t* = 3.51, p__adj_ = 0.003, Cohen’s d = 0.91), with this reduction sustained at Week 12 (Baseline vs Week 12: *t* = 3.34, p__adj_ = 0.004, Cohen’s d = 0.85). In contrast, the reduction in sensory hyperresponsiveness (Baseline vs. Week 12: *t* = 2.77, p__adj_ = 0.021, Cohen’s d = 0.71) was significant only at Week 12. Sensitivity analyses by excluding the two participants with suboptimal compliance confirmed that all significant results were maintained (Supplementary Table 2).

### Gut microbiota alterations and associations with symptom improvement

**Fig. 3:**
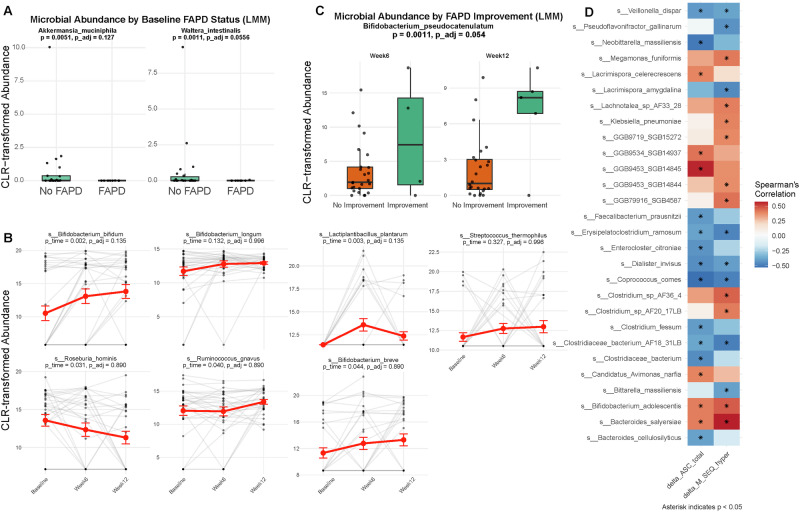
Changes in fecal microbiota and their association with clinical outcomes in children with ASD. **A** Partial Spearman correlations (adjusted for age, sex, BMI, total energy intake, medication use, and ADHD diagnosis) between baseline microbial abundances and functional abdominal pain disorders (FAPD). **B** Linear mixed models (LMM) identifying bacterial species with significant changes in relative abundance over the 12-week SCM06 treatment, adjusted for repeated measures. **C** Association between change in *Bifidobacterium pseudocatenulatum* abundance and improvement in FAPD (LMM, p__adj_ = 0.054). p__adj_: Benjamini-Hochberg adjusted p-value. **D** Fecal bacterial species that correlated with changes in anxiety (delta_ASC_total) and sensory hyperresponsiveness (delta_M_SEQ_hyper) symptoms. FAPD Functional Abdominal Pain Disorders. M_Seq_hyper Sensory hypersensitivity. ASC Anxiety symptoms.

At baseline, partial Spearman correlations adjusting for age, sex, BMI, total energy intake, medication use, and ADHD diagnosis showed that *Waltera intestinalis* (rho = −0.624, *p* = 0.001, p__adj_ = 0.056) and *Akkermansia muciniphila* (rho = −0.553, p = 0.005, p__adj_ = 0.127) were negatively correlated with functional abdominal pain (Fig. 3A). LMM was performed to identify microbiome alteration following SCM06 treatment. The relative abundance *of Bifidobacterium bifidum* and *Lactobacillus plantarum* increased (p < 0.01, p__adj_ = 0.135, LMM, Fig. 3B). There was also an trend towards increased abundance of *Bifidobacterium longum* and *Streptococcus thermophilus* (*p* > 0.05, LMM, Fig. 3B). Besides species contained in SCM06, changes in the relative abundances of *Roseburia hominis, Ruminococcus gnavus* and *Bifidobacterium breve* were associated with SCM06 treatment (*p* < 0.05, p__adj_ > 0.25, Fig. 3B). However, there was no changes in species level alpha diversity between time points (Supplementary Fig. 1A). PERMANOVA stratified by subject revealed no significant association between gut microbiota composition and time, age, sex, BMI, or changes in clinical symptoms (*p* > 0.05) (Supplementary Fig. 1B, C). Following SCM06 treatment, increase in *Bifidobacterium pseudocatenulatum* was associated with improved functional abdominal pain disorders (*p* = 0.0011, p__adj_ = 0.054, LMM, Fig. 3C).

To investigate the relationship between clinical improvements and microbiota alterations, we performed correlation analysis between changes in clinical symptoms and clr-transformed abundances from Baseline to Week 12 following synbiotic treatment. Although no associations survived FDR correction (all p__adj_ > 0.25), nominally significant correlations were observed between improvements in anxiety and sensory hyperresponsiveness and changes in multiple species. Increase of *Faecalibacterium prausnitzii* correlated with reduction in anxiety after 12 weeks of SCM06 treatment (*p* = 0.028, *r* = −0.414, Spearman’s test, Fig. 3D). On the other hand, increase in *Clostridiaceae bacterium AF18 31LB, Coprococcus comes, Dialister invisus, Erysipelatoclostridium ramosum*, and *Veillonella dispar* were associated with improvements in both anxiety and sensory hyperresponsiveness (all *p* < 0.05, Spearman’s test, Fig. 3D).

### Changes in fecal metabolome and associations with symptom improvement

**Fig. 4:**
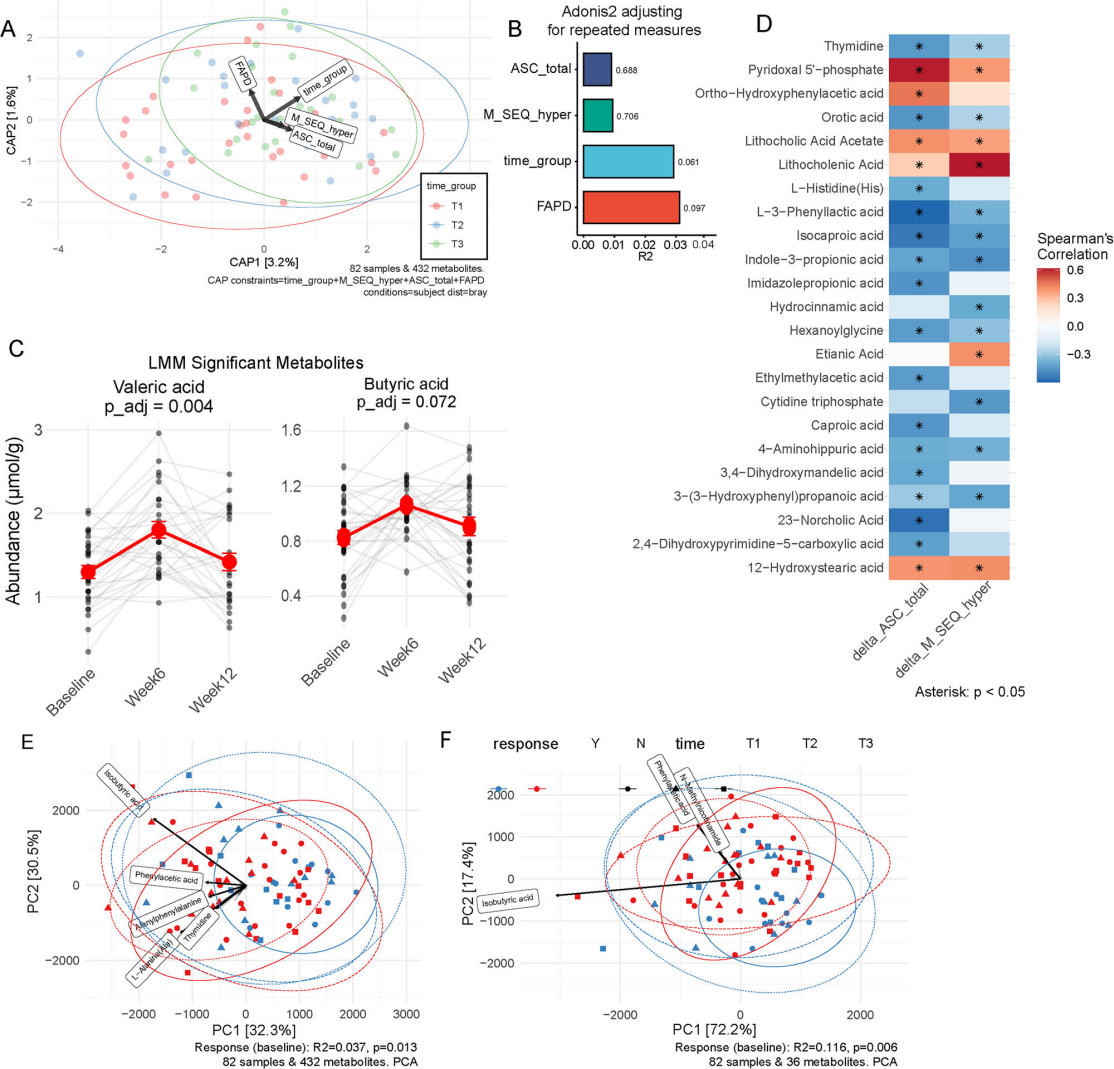
Fecal metabolome in children with ASD before and after synbiotic treatment. **A** Constrained partial coordination plot for change in fecal metabolome and improvement in clinical symptoms. **B** Impact of physical, clinical symptoms, fecal metabolome throughout the SCM06 treatment course, adjusting for repeated measures. **C** Line plots depicting levels of fecal metabolotes changed following SCM06 treatment. **D** Fecal metabolites that correlated with changes in anxiety (delta_ASC_total) and sensory hyperresponsiveness (delta_M_SEQ_hyper) symptoms. **E** PCA plot of overall metabolome profile in responders and non-responders. **F** PCA plot of metabolites showing significant changes following SCM06 intervention in responders and non-responders. FAPD Functional Abdominal Pain Disorders. M_Seq_hyper Sensory hypersensitivity. ASC Anxiety level.

For fecal metabolomic profiling, a total of 432 metabolites were identified with absolute quantification, majority of which were related to amino acids, carbohydrates, and lipid metabolism (Supplementary Fig. 2). Although symptoms of abdominal pain, anxiety, and sensory hyperresponsiveness did not reach statistical significance in explaining overall metabolome variance (*p* > 0.05), PERMANOVA analysis revealed a marginal shift in overall metabolome after treatment (pseudo-F = 2.05, R² = 0.029, *p* = 0.061, Fig. 4A & B). The abundances of valeric acid and butyric acid increased following SCM06 treatment (p__adj_ = 0.004 and p__adj_ = 0.072, respectively, LMM, Fig. 4C). In addition, several metabolites, including N-Acetylserotonin (NAS), a key intermediate in the melatonin biosynthesis pathway and a metabolite of serotonin, increased following SCM06 treatment (*p* = 0.039, p__adj_ > 0.25, Supplementary Fig. 3).

Similar to compositional changes, no associations survived FDR correction (p__adj_ > 0.25). Nominally significant correlations were observed between improvements in anxiety and sensory hyperresponsiveness and changes in multiple metabolite classes, including bile acids, carbohydrates, amino acids, and fatty acids (all *p* < 0.05, Fig. 4D). Specifically, reduced levels of 12-Hydroxystearic acid were associated with improvement of both anxiety (*r* = 0.39, *p* = 0.048) and sensory hyperresponsiveness (*r* = 0.41, *p* = 0.040). Similarly, decreased lithocholic acid acetate correlated with reduced anxiety (*r* = 0.40, *p* = 0.041), while lower lithocholenic acid strongly correlated with improved sensory hyperresponsiveness (*r* = 0.62, *p* = 0.0008). In contrast, increased levels of indole-3-propionic acid (anxiety: *r* = −0.42, *p* = 0.035; sensory hyperresponsiveness: *r* = −0.47, *p* = 0.015) and isocaproic acid (anxiety: *r* = −0.54, *p* = 0.004; sensory hyperresponsiveness: *r* = −0.43, *p* = 0.029) were associated with improvement of both anxiety and sensory hyperresponsiveness.

Next, subjects were classified as responders and non-responders based on the mean changes in the ASC-ASD total score (a reduction ≥ 5), and the Hyperresponsiveness subscale score of the SEQ (a reduction ≥ 0.3) from baseline to Week 12. Significant differences were observed between responders and non-responders in both the baseline overall metabolome profile (R^2^ = 0.037, *p* = 0.013, Fig. 4E) and among metabolites showing significant changes following SCM06 intervention (R^2^ = 0.116, *p* = 0.013, Fig. 4F, Supplementary Fig. 4A). Following intervention, the fecal metabolome of responders demonstrated a marked shift that was absent in non-responders (Fig. 4F, Supplementary Fig. 4B). This shift was primarily driven by changes in isobutyric acid and phenylactic acid (Fig. 4E, F).

### Changes in microbial metabolic pathways and association with symptom improvement

LMM identified five pathways with nominal changes over time (with *p* < 0.05, yet all p__adj_ > 0.25, Supplementary Fig. 5A). These included pathways involved in pyrimidine metabolism (e.g., PWY-7185: UTP and CTP dephosphorylation I, *p* = 0.0096; PWY-7210: pyrimidine deoxyribonucleotides biosynthesis from CTP, *p* = 0.0131) and sucrose degradation (PWY-7345, *p* = 0.0212). Similar to fecal metabolome, no associations survived FDR correction (all p__adj_ > 0.25), nominally significant correlations were observed between improvements in anxiety and sensory hyperresponsiveness that may have potential link with compositional and metabolomic findings (Supplementary Fig. 5B). Increase in fermentation-related pathways, including pyruvate to butanoate fermentation (*r* = −0.42, *p* = 0.027), mixed acid fermentation (*r* = -0.4, *p* = 0.035) and superpathway of *Clostridium acetobutylicum* acidogenic fermentation (*r* = −0.43, *p* = 0.023) were correlated with improvements in anxiety. On the other hand, improvement of sensory hyperresponsiveness was associated with decrease in L-histidine biosynthesis (*r* = 0.42, *p* = 0.028) and peptidoglycan biosynthesis pathways (*r* = 0.41, *p* = 0.031). In addition, functional abdominal pain improvement was associated with increase in homolactic fermentation (*r* = −0.43, *p* = 0.021), acetyl-CoA to butanoate fermentation (*r* = −0.42, *p* = 0.026), succinate to butanoate fermentation (*r* = −0.38, *p* = 0.049), and *Bifidobacterium* shunt (*r* = −0.40, *p* = 0.036), as well as decrease in L-citrulline biosynthesis (*r* = 0.41, *p* = 0.030) and the urea cycle (*r* = 0.46, *p* = 0.015). However, the GABA shunt pathway did not change significantly after intervention, nor was it associated with change in the clinical symptoms (Supplementary Fig. 6).

### Mediation analysis reveals bacterial species as key mediators of clinical improvements

**Fig. 5:**
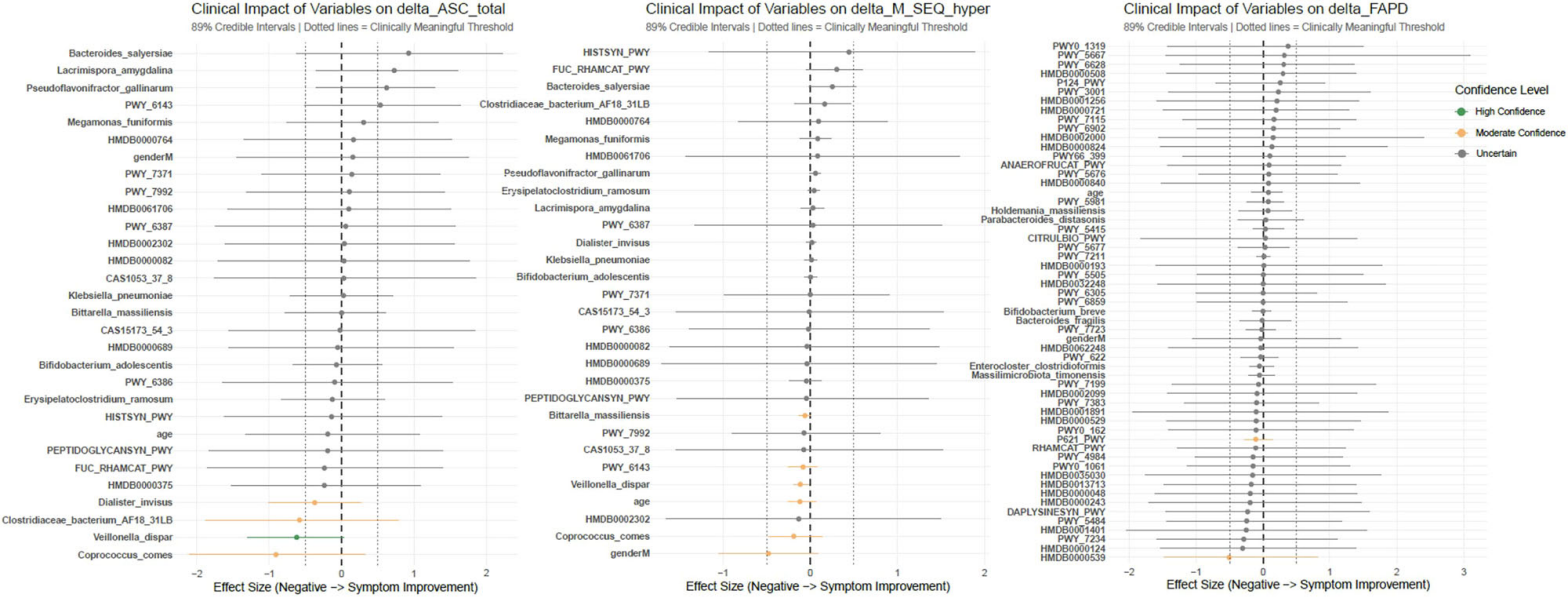
Mediation analysis of fecal microbial species, metabolic pathways and metabolites on clinical changes in anxiety (detla_ASC_total), sensory hyperresponsiveness (delta_M_Seq_hyper) and functional abdominal pain (delta_FAPD).

Exploratory mediation analysis was performed to investigate whether the observed clinical improvements (reduction in severity of anxiety and sensory hyperresponsiveness) were directly driven by synbiotic treatment or indirectly mediated by changes in gut microbiota (taxonomy or metabolome). The analysis identified *Coprococcus comes* and *Veillonella dispar* as candidate species with high probability ( > 0.7) of contributing to symptom changes (Fig. 5). No significant candidate mediation was observed for metabolic pathways or metabolites.

## Discussion

To our knowledge, this is the first study to show that a newly developed synbiotic preparation is safe and was associated with reductions in parent-reported anxiety and sensory hyperresponsiveness in children with ASD. Most previous studies of microbiota modulation in ASD that focused on global symptoms without clearly defined domains of targeted symptoms have yielded mixed results^13^. The mild and transient nature of gastrointestinal side effects, minimal dropouts, as well as the ease of administration suggest that SCM06 may be a promising treatment option for these children, and larger studies are needed to confirm these findings. Explorative findings in the gut microbiota and metabolomic profile allude to possible mechanisms of therapeutic action that warrants further investigations.

The heterogeneity of symptoms and severity in ASD suggests that intervention strategies need to be individualised for specific symptoms. The present study is distinguished from existing studies of microbiota-modulation therapy through several key features. First, we developed a synbiotic formula containing four probiotic species, including *Bifidobacterium bifidum, Bifidobacterium longum, Lactobacillus plantarum*, and *Streptococcus thermophilus*, which are known for their ability to enhance GABA production and regulate the immune system in laboratory settings^16–19^, to target the postulated pathways within the microbiota-gut-brain axis specific to sensory hyperresponsiveness and anxiety symptoms in ASD. Our in vitro analysis also showed that this synbiotic can induce GABA production (data on file). Secondly, we selected a well-defined group of children with ASD characterized by elevated levels of anxiety and sensory hyperresponsiveness. Approximately one third of the children screened fulfilled the criteria, which aligned with the reported prevalence of anxiety in children with ASD^1^. Our study population was representative, as their mean scores in the core ASD symptoms (SCI and RRB of the SRS-2), and other co-occurring psychopathology (subscales of the CBCL) were all above the cut-offs at baseline^20,21^.

We also found that the prevalence of children with functional abdominal pain decreased from 26.7% to 10.0% over the 12-week course of SCM06. Co-occurrence of functional GI symptoms in children with ASD is known to be associated with more severe clinical symptoms of ASD^22–25^, as well more severe alterations in the gut microbiota composition^26^. Notably, the pathophysiology of functional GI disorders varies by subtype^27^, and central and visceral sensory dysfunctions were thought to be key mechanisms for functional abdominal pain^28^. As microbiota modulation was observed to reduce sensory dysfunctions in patients with irritable bowel syndrome^29^ and functional abdominal pain is also more likely than other functional GI disorders to be associated with anxiety symptoms in children with ASD^25,30,31^, we propose that SCM06 may alleviate functional abdominal pain and anxiety through a shared mechanism within the microbiota-gut-brain axis.

Given the small sample size of this pilot study, most of our metagenomic and metabolomic analyses did not survive FDR correction at a stringent threshold of p__adj_ < 0.25 after controlling for multiple comparisons. However, these exploratory analyses are informative for hypothesis generation regarding possible mechanisms that warrant further investigation. We discuss these nominal trends (*p* < 0.05) and report both nominal and adjusted p-values for transparency in our exploratory metagenomic and metabolomic results. Contrary to our initial postulation, the GABA shunt pathway did not change significantly after SCM06 treatment, nor was it associated with symptom improvements. This prompted a shift toward other nominal changes, which may allude to alternative mechanisms. For instance, improvements in anxiety and sensory hyperresponsiveness were associated with increases in butyrate-producing gut bacteria, such as *Coprococcus comes* and *Faecalibacterium prausnitzii*^32,33^. Functional shifts in butyrate-generating fermentation pathways were also linked to anxiety symptoms and functional abdominal pain disorders improvement. These taxonomic and functional alterations paralleled increases in fecal SCFA levels, including butyric acid and valeric acid. Such changes may contribute to clinical improvement through SCFA-dependent mechanisms. First, butyrate serves as a histone deacetylase (HDAC) inhibitor, which potentially enhances GABAergic function and balances the excess excitatory neurotransmission often seen in ASD^34,35^. Second, butyrate and propionate exert anti-inflammatory effects by suppressing microglial activation and reducing pro-inflammatory cytokines, which are known to exacerbate sensory hyperresponsiveness^36,37^. Third, SCFAs can directly modulate gut-brain communication via vagus nerve stimulation or by strengthening intestinal barrier integrity, thereby lowering systemic endotoxin exposure and its downstream neuroinflammatory effects^38^. The increase in propionate-producing *Veillonella dispar* further supports this mechanism, as propionate activates free fatty acid receptors (FFAR3) to modulate sensory processing^39,40^. The combined effects of butyrate and propionate may thus help restore the excitatory-inhibitory balance in neural circuits, reducing sensory hyperresponsiveness.

Notably, we observed a nominal increase in fecal NAS, a key intermediate in the melatonin biosynthesis pathway, which may exert potential neuroprotective effects through its antioxidant properties^41^. The association between decreased L-histidine biosynthesis and improvement of sensory hyperresponsiveness may reflect the physiological effect of histamine production, which enhances glutamatergic transmission in sensory pathways^42,43^ Although the GABA shunt pathway remained unchanged, alternative routes for GABA modulation may exist, including butyrate-induced glutamic acid decarboxylase (GAD) expression^44^ and indole-3-propionic acid’s aryl hydrocarbon receptor (AhR)-mediated anti-inflammatory effects on cortical circuits^45^.

The exploratory mediation analysis revealed that *Coprococcus comes* and *Veillonella dispa*r, both SCFA producers, are candidate mediators of the improvements in ASD-related symptoms following synbiotic treatment. Notably, while the fecal metabolome showed nominal changes after treatment, no individual metabolites or pathways emerged as mediators in the formal analysis. This possibly indicates that the bacteria may also influence host function through non-metabolic pathways, such as immune modulation or direct neural signaling^35^.

This study has several limitations. First, the small sample size and open-label design suggest that a larger randomized placebo-controlled study is needed to confirm its efficacy, as well as verifying the results of the bioinformatic analyses. Second, the study duration was only 12 weeks and whether symptom improvement can be sustained beyond this period is unclear, though we believe that safety is unlikely to be an issue with longer-term therapy. Third, there was no placebo control group and outcome evaluation was based on unblinded parents’ observations without an independent assessment, which could be confounded by placebo effect or natural course of the disorder, making it difficult to assess the effect of the synbiotic. Furthermore, we did not capture sleep and physical activities, which may also confound the effect of SCM06. While we have controlled for RTM in our statistical analysis, the observed symptom improvements, as well as the effect sizes, should be conservatively interpreted and adjusted when designing a powered trial. Fourth, the biological mechanisms underlying SCM06’s therapeutic effects, including its impact on the microbiota-gut-brain axis, remain speculative and should be determined through experimental and pre-clinical studies. In particular, targeting *Coprococcus* and *Veillonella*, such as gnotobiotic colonization experiments or fecal microbiota transplantation studies in ASD models will shed light on the development of novel live biotherapeutics for ASD.

In conclusion, SCM06 is well-tolerated, safe, and shows potential efficacy for anxiety and sensory hyperresponsiveness in children with ASD. Modulation of the gut microbiome offers a novel therapeutic avenue, but deeper mechanistic understanding of the microbiota-gut-brain axis is crucial in optimizing microbiome modulation therapies for ASD. Effective ASD management must account for clinical heterogeneity, tailoring interventions to specific symptomatology and selecting appropriate candidates to maximize impact. Larger, randomized controlled trials and mechanistic studies are needed to confirm SCM06’s efficacy and guide its integration into personalized ASD treatment strategies.

## Methods

This was an open-label pilot study to examine the safety, tolerability, and efficacy of a 12-week course of SCM06 in treating anxiety and sensory hyperresponsiveness in children with ASD. Parents or guardians of participating children provided written informed consent in accordance with the Declaration of Helsinki and the Guideline for Good Clinical Practice of the International Council for Harmonisation of Technical Requirements for Pharmaceuticals for Human Use (ICH-GCP). Ethics approval was granted by the Joint CUHK-NTEC Clinical Research Ethics Committee (reference: 2023.484). This clinical trial was registered at ClinicalTrials.gov ID: NCT06126185.

### Study population

Chinese children with ASD aged 12 years or younger were recruited from a cohort established by our team between 2021 and 2023^11,46^. Participants of this cohort were recruited from a university-affiliated tertiary child and adolescent psychiatry specialist clinic in Hong Kong, which serves a catchment area of over 1.2 million people. The clinic receives referrals from developmental pediatricians, clinical and educational psychologists, and private general practitioners for the assessment, training, and treatment of children with neurodevelopmental conditions. The diagnosis of ASD was made by child psychiatrists according to the Diagnostic and Statistical Manual of Mental Disorders, Fifth Edition (DSM-5) diagnostic criteria^47^. Inclusion criteria included significant anxiety, defined as a total score of 20 or higher on the parent-rated Anxiety Scale for Children-ASD (ASC-ASD)^48^, and significant sensory hyperresponsiveness, defined as the mean score of 2.5 or greater in the Hyperresponsiveness Subscale of the Sensory Experiences Questionnaire (SEQ)^49^. Exclusion criteria included intellectual disability, neurological disorders, psychosis, depressive disorders, current use of antidepressants, use of probiotics or antibiotics within 4 weeks, adherence to a special diet (e.g., vegetarian), organic GI disorders (e.g., inflammatory bowel disease and Hirschsprung’s disease), known allergies to SCM06 ingredients, or other major medical illnesses. An estimate of precision using a confidence interval was used to determine sample size of this pilot study. Based on standard deviations (SD) from our local cohort of children with ASD (SD = 9.89 for ASC-ASD total score, SD = 0.53 for SEQ Hyperresponsiveness subscale)^25^, a sample size of 30 provides a half-width 95% confidence interval of approximately 3.54 units for the mean change in anxiety and 0.19 units for sensory hyperresponsiveness, which are about 36% of the respective SDs and narrow enough to account for natural variability.

### Procedures

From December 2023 to July 2024, investigators screened for eligible participants. As part of the longitudinal study, parents or caregivers completed the ASC-ASD and SEQ for their participating child, and investigators evaluated eligibility based on the inclusion and exclusion criteria. The research team approached parents or caregivers of potential participants to explain the study details. After obtaining informed consent from the parents or caregivers, the baseline assessment was conducted as follows: Sociodemographic data were collected from the parent or caregiver, and medical records were reviewed for co-occurring psychiatric diagnoses and use of psychiatric medications (stimulants or antipsychotics). Participants were measured for body height, weight, and body mass index (BMI). Clinical characteristics were assessed using parent-rated questionnaires, including the ASC-ASD, sensory hyporesponsiveness and hyperresponsiveness subscales of the SEQ, the Chinese version of the Social Responsiveness Scale, Second Edition (SRS-2) to assess the core symptoms of ASD^20^, and the Child Behavior Checklist (CBCL), which provides three subscales for Attention Problems, Internalizing, and Externalizing Problems^21,50^. Functional Gastrointestinal Disorders (FGIDs) were assessed with the Rome IV Diagnostic Questionnaires for Pediatric FGID: Parent report form for Children (R4PDQ), which identifies 10 Rome IV-defined FGIDs grouped into three main categories of Functional Nausea and Vomiting Disorders (FNVD), Functional Abdominal Pain Disorders (FAPD), and Functional Defecation Disorders (FDD)^51^. The R4PDQ was validated in community-based school children against diagnosis by gastroenterologists^52^ and was translated into Chinese with approval from the Rome Foundation Committee^30^. Participants’ diet was evaluated using the 3-day dietary food record (3DFR)^53^. Foodworks nutrition analysis software (Xyris Software, Australia) was used to estimate daily macronutrient consumption (carbohydrates, fat and protein) in grams and total energy intake in kcal. For each participant, the averaged dietary intake throughout the 12-week course of SCM06 were compared against the Chinese Dietary Reference Intakes (DRI, standard code WS/T 578)^54^ to identify dietary problems, defined as any of the following: 1. Abnormal energy intake, defined as energy intake fell below 75% or exceeded 125% of the estimated energy requirement (EER), 2. Carbohydrate insufficiency, 3. Protein insufficiency, 4. Fat underconsumption and overconsumption. A binary variable for the presence of any dietary problems was computed. Two baseline stool samples were collected by parents or caregivers at home with guidance from the research team: one in a tube containing Norgen preservatives (cat. 63700, Norgen Biotek Corp, Ontario, Canada) for metagenomics and the other with OMNImet®•GUT (DNA Genotek Inc) for metabolomics study. The stool samples were delivered to the laboratory within 24 h of collection and stored at −80 °C after aliquoting. All fecal samples were processed in the laboratory as a single batch for both metagenomics and metabolomics upon completion of the trial.

Following the baseline assessment, participants commenced a 12-week daily course of SCM06. Parents were instructed to mix the SCM06 formula into water, juice, or milk for the participant’s consumption. Follow-up visits were conducted at Week 6 and Week 12. At each follow-up visit, compliance with the synbiotic formula was assessed by counting used and unused SCM06 packages. Tolerability was assessed using a standardized questionnaire at both follow-up visits to screen for GI side effects, including diarrhoea, abdominal pain, reduced appetite, nausea and vomiting, fever, rash, headache, mood changes, and any other symptoms. The research team, including study clinicians, discussed reported symptoms with the parent or caregiver to determine their potential relation to SCM06. The ASC-ASD, SEQ, SRS-2, CBCL, R4PDQ, 3DFR were readministered to the same parent or caregiver to monitor changes in clinical symptoms and diet. Body weight and height were measured, and two stool samples were collected at each follow-up. Parents or caregivers were requested to avoid initiating any new treatment or training programme during the 12-week study period, unless clinically necessary. Any changes in clinical management, including new medications, dosage adjustments, training programs, or psychological interventions, were inquired about and reviewed in the medical records.

### Outcomes

The primary outcome was the safety and tolerability of the SCM06. Secondary outcomes included changes in anxiety and sensory hyperresponsiveness symptoms at week 12. Additionally, we explored changes in core ASD symptoms, externalizing and internalizing behaviours, ADHD symptoms, FGID symptoms, and gut metagenomics and metabolomics.

### Statistical analysis

Descriptive statistics, including counts, means or medians, SD, interquartile ranges, and percentages, were used to summarize the clinical characteristics of the study population and the safety and tolerability of SCM06. Linear mixed models (LMMs) were fitted to primarily assess changes in anxiety (total score of ASC-ASD), sensory hyperresponsiveness (mean score of the Sensory Hyperresponsiveness subscale of SEQ), and other continuous clinical scores from baseline to Week 6 and Week 12. Each model included the respective baseline score, time, and a baseline-by-time interaction to evaluate RTM. Age, sex, BMI, 12-week average daily total energy intake, presence of dietary problems, psychiatric medication use, co-occurring ADHD were included as covariates. Random intercepts for subjects accounted for within-subject correlation. Type III ANOVA with Satterthwaite approximation tested the overall time effect, with partial eta squared (ηp2) as the effect size. Model comparisons using likelihood ratio tests assessed the significance of the baseline-by-time interaction for RTM. Tukey-adjusted pairwise contrasts examined specific timepoint differences. Changes in the prevalence of FGIDs across the 12 weeks were explorative in nature evaluated with Cochran’s Q test with False Discovery Rate (FDR) correction for multiplicity. A threshold of alpha ≤ 0.05 was considered statistically significant.

### Faecal microbial DNA extraction

Faecal microbial DNA was isolated with DNeasy PowerSoil Pro Kit (Qiagen, Hilden, Germany) according to manufacturer’s instructions. Briefly, Solution CD1 was added to the faecal samples. To lyse cells, the samples were incubated at 65 °C for 10 min and bead-beat at 25 Hz for 10 min using TissueLyser II. After that, the samples were centrifuged at 15,000 × *g* for 1 min. Solution CD2 was then added to the clear supernatant to remove PCR inhibitors. The samples were centrifuged again, and the supernatant was transferred to another tube where Solution CD3 was added. The mixture was loaded to the MB spin column to bind DNA in the filter membrane. Membrane-bound DNA was washed with Solution EA and C5 and finally eluted into another 2 mL tube. DNA quality was assessed using Qubit Fluorometer and agarose gel electrophoresis. Qualified DNA samples were stored at −80 °C for further library construction.

### Shotgun metagenomics sequencing and profiling

Sequencing libraries were prepared from extracted DNA using the Illumina DNA Prep (Illumina, California, USA). After quality control procedures by Qubit Fluorometer and Agilent 2100 Bioanalyzer, the DNA libraries were sequenced on Illumina NovaSeq 6000 System, yielding an average of 16 ± 4.3 million reads per sample. Raw sequence reads were quality-trimmed using fastp v0.23.2 to remove low-quality bases with default parameters. Host reads were filtered using Bowtie2 v2.4.5 aligned to the CHM13 human genome database. Taxonomic profiling was performed using MetaPhlAn v4.0.6 with the mpa_vOct22_CHOCOPhlAnSGB_202212 database. Functional profiling was conducted using HUMAnN v3.8, which included annotation of species pan-genomes via the CHOCOPHLAN V201901_V31 database.

### Metabolomic profiling

Approximately 100 mg of stool sample from each OMNImet®•GUT tube was homogenized with deionized water and release agent using a mechanical grinder (JXFSTPRP, 50 Hz, 300 s) followed by centrifugation (18,000 × g, 4 °C, 20 min). The supernatant was derivatized with reagent working solution (40 °C, 1200 rpm, 60 min), diluted with pre-cooled solvent, and centrifuged (4000 × g, 4 °C, 5 min) prior to analysis. Metabolite profiling was performed using a Waters UPLC I-Class Plus system coupled to a SCIEX QTRAP 6500 Plus mass spectrometer. Separation was achieved on a BEH C18 column (2.1 × 100 mm, 1.7 μm) with a gradient of 0.1% formic acid in water and acetonitrile containing 30% isopropanol at 0.4–0.6 mL/min and 40°C. Mass spectrometry was conducted in MRM mode with electrospray ionization ( ± 4500 V, 400 °C) using gas pressures of 60 psi (GS1/GS2) and 35 psi (curtain gas). Metabolite identification and quantification were performed using Skyline software (v21.1.0.146) with a mass range of 50–1500 Da and 0.6 Da tolerance. All solvents were LC-MS grade (Thermo Fisher Scientific, Honeywell, DIMKA) and ultrapure water was generated using a Milli-Q Integral system.

### Bioinformatics analysis

Beta-diversity differences were assessed using PERMANOVA on Aitchison distances with subject adjustment for repeated measures. We tested the effects of clinical variables (ASC-ASD and Sensory Hyperresponsiveness Subscale of SEQ) and Time (Baseline/Week 6/Week 12) using marginal effects with 999 permutations. Baseline associations between microbial species abundance and symptom severity were evaluated using partial Spearman correlations, adjusting for age, sex, BMI, total energy intake, medication use, and ADHD diagnosis. Longitudinal changes in species, pathways and metabolites were analyzed using LMM with CLR-transformed abundance for species and pathways as outcome, including time point, demographic and clinical covariates as fixed effects, and subject-specific random intercepts. Time effects were evaluated by Type III ANOVA with Benjamini-Hochberg FDR correction (significance threshold p_adj < 0.25). Nominal and adjusted p-values are reported given the exploratory nature and small sample size. Bayesian multiple regression (brms) was employed using MaAsLin2-pre-selected features (*p* < 0.05) as predictors of clinical outcomes, with age and gender as fixed effects and subject random intercepts. Weakly informative priors provided regularization, while leave-one-out cross-validation and MCMC diagnostics (Rhat and effective sample size) ensured model robustness.

## Supplementary information

Supplementary Information

## Data Availability

Sequencing data were uploaded to NCBI under Bioproject PRJNA1274164 (https://www.ncbi.nlm.nih.gov/bioproject/?term=PRJNA1274164). Participant metadata cannot be made publicly available via repositories as outlined in the patient consent form to protect participant privacy. Requests for sharing metadata can be submitted with a written proposal to the corresponding author (Prof. Siew C. Ng) at siewchienng@cuhk.edu.hk.
