# Supplementary material for: A novel synbiotic (SCM06) for anxiety and sensory hyperresponsiveness in children with autism spectrum disorder: an open-label pilot study
Source: NPJ Biofilms Microbiomes. 2026 Jan 14;12:36. doi: 10.1038/s41522-025-00902-8 (PMC12881606; doi:10.1038/s41522-025-00902-8)
Supplement: Supplementary file 1 — Supplementary Information [file 41522_2025_902_MOESM1_ESM.docx]

**Supplementary Materials**

**A novel synbiotic (SCM06) for anxiety and sensory hyperresponsiveness in children with autism spectrum disorder: an open-label pilot study**

**Content**

| **Supplementary Table 1**  **Supplementary Table 2** | Average daily dietary intake of the participants over the 12-week course of SCM06  Sensitivity analysis of the main significant findings by removing two subjects with suboptimal compliance to the synbiotic formula |
| --- | --- |
| **Supplementary Figure 1** | a) Changes in species alpha diversity throughout the 12-week course of SCM06. b) Beta diversity analysis across Baseline (T1), Week 6 (T2) and Week 12 (T3). c) Overall community composition throughout the 12-week course of SCM06 at genus level. |
| **Supplementary Figure 2** | Classification of all detected 432 metabolites with absolute quantification |
| **Supplementary Figure 3** | Fecal metabolites that showed nominal changes during SCM06 treatment (p < 0.05, linear mixed models). |
| **Supplementary Figure 4** | a) Heatmap comparing the abundance of fecal metabolites between responders and non-responders. Only metabolites that exhibited significant changes following SCM06 intervention were shown. b) Overall fecal metabolome shifts in responders and non-responders. |
| **Supplementary Figure 5** | a) Metabolic Functional Pathways that showed nominal changes during SCM06 treatment (p < 0.05, linear mixed models) b) Metabolic Functional Pathways that correlated with changes in anxiety (delta_ASC_total), sensory hyperresponsiveness (delta_M_SEQ_)hyper) symptoms, and functional abdominal pain disorders (delta_FAPD). |
| **Supplementary Figure 6** | a) Changes in the GABA Shunt pathway throughout the 12-week course of SCM06. b) Correlation of the GABA Shunt pathway and changes in anxiety (delta_ASC_total) and sensory hyperresponsiveness (delta_M_SEQ_hyper) symptoms. |

|  |  |
| --- | --- |

| **Supplementary Table 1.** | | | |
| --- | --- | --- | --- |
| Average daily dietary intake of the participants over the 12 weeks of SCM06 | | | |
|  |  | Mean/n | SD/% |
| Energy (kcal) | | 1974.3 | 446.4 |
| Estimated energy requirement (EER) | |  |  |
|  | < 75% EER | 1 | 3.3 |
|  | 75 ~ 125% EER | 22 | 73.3 |
|  | > 125% EER | 7 | 23.3 |
| Carbohydrate (g) | | 262.2 | 54.9 |
|  | Carbohydrate insufficiency | 4 | 13.3 |
| Protein (g) | | 87.5 | 23.8 |
|  | Protein insufficiency | 2 | 6.7 |
| Fat (g) | | 60.3 | 20.6 |
|  | Fat underconsumption | 1 | 3.3 |
|  | Fat overconsumption | 4 | 13.3 |
| Note: The Chinese dietary reference intakes (DRIs) was reference for the estimated energy requirement (EER), carbohydrate and protein insufficiency, as well as far underconsumption and overconsumption. | | | |

| **Supplementary Table 2.** | | | | | | | |  |  |  |  |  |  |  |  |  |  |  |  |  |
| --- | --- | --- | --- | --- | --- | --- | --- | --- | --- | --- | --- | --- | --- | --- | --- | --- | --- | --- | --- | --- |
| Sensitivity analysis of the main significant findings by removing two participants with suboptimal compliance to the synbiotic formula | | | | | | | | | | | | | | | | | | | | |
| Symptoms | Baseline | Week 6 | Week 12 |  | Statistics | | | |  | Baseline vs Week 6 | | |  | Baseline vs Week 12 | | |  | Week 6 vs Week 12 | | |
|  | Mean/n (SD/%) | Mean/n (SD/%) | Mean/n (SD/%) |  | F/Q | df | p | ηp2 |  | t | p_Tukey_ | d |  | t | p_Tukey_ | d |  | t | p_Tukey_ | d |
| ASC-ASD Total Score | 36.60 (11.00) | 31.40 (12.70) | 31.50 (11.10) |  | 6.46 | 2, 52 | 0.003** | 0.199 |  | 3.13 | 0.008** | 0.84 |  | 3.09 | 0.009** | 0.83 |  | -0.07 | 0.999 | -0.01 |
| SEQ Hyperresponsiveness Subscale | 3.25 (0.51) | 3.11 (0.65) | 2.60 (0.77) |  | 3.50 | 2, 52 | 0.038* | 0.118 |  | 1.58 | 0.262 | 0.42 |  | 2.63 | 0.030* | 0.70 |  | 1.05 | 0.552 | 0.28 |
| FAPD | 8 (40) | 4 (20) | 3 (15) |  | 8.4 | 2 | 0.045* |  |  | - | | | | | | | | | | |
| *Note*: * p < 0.05, ** p < 0.01, ASC-ASD: Anxiety Scale for Children - ASD; SEQ: Sensory Experiences Questionnaire; FGID: Functional Gastrointestinal Disorder; FAPD; Functional Abdominal Pain Disorders | | | | | | | | | | | | | | | | | | | | |

**
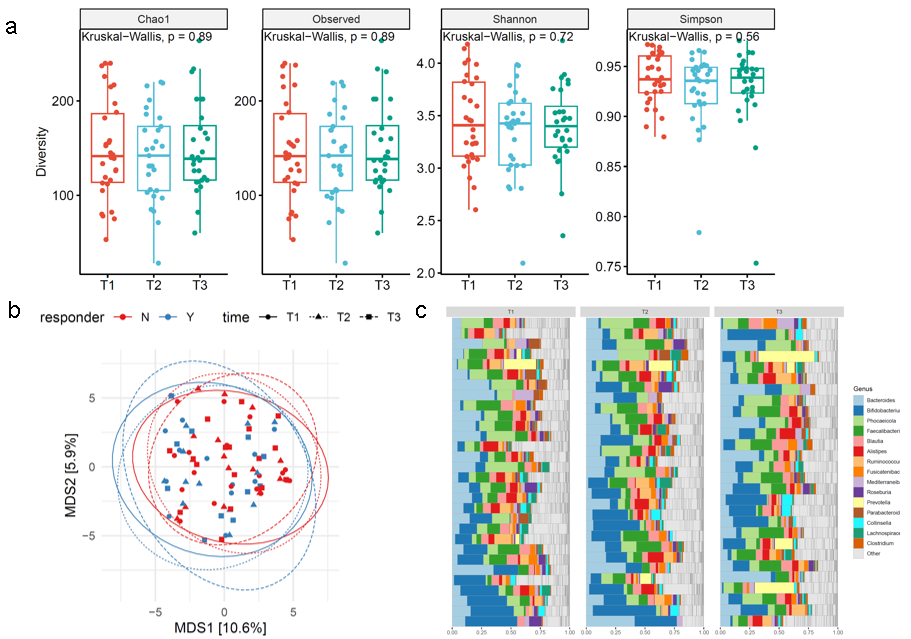
**

**Supplementary Figure 1.** a) Changes in species alpha diversity throughout the 12-week course of SCM06. b) Beta diversity analysis across Baseline (T1), Week 6 (T2) and Week 12 (T3). c) Overall community composition throughout the 12-week course of SCM06 at genus level.

**
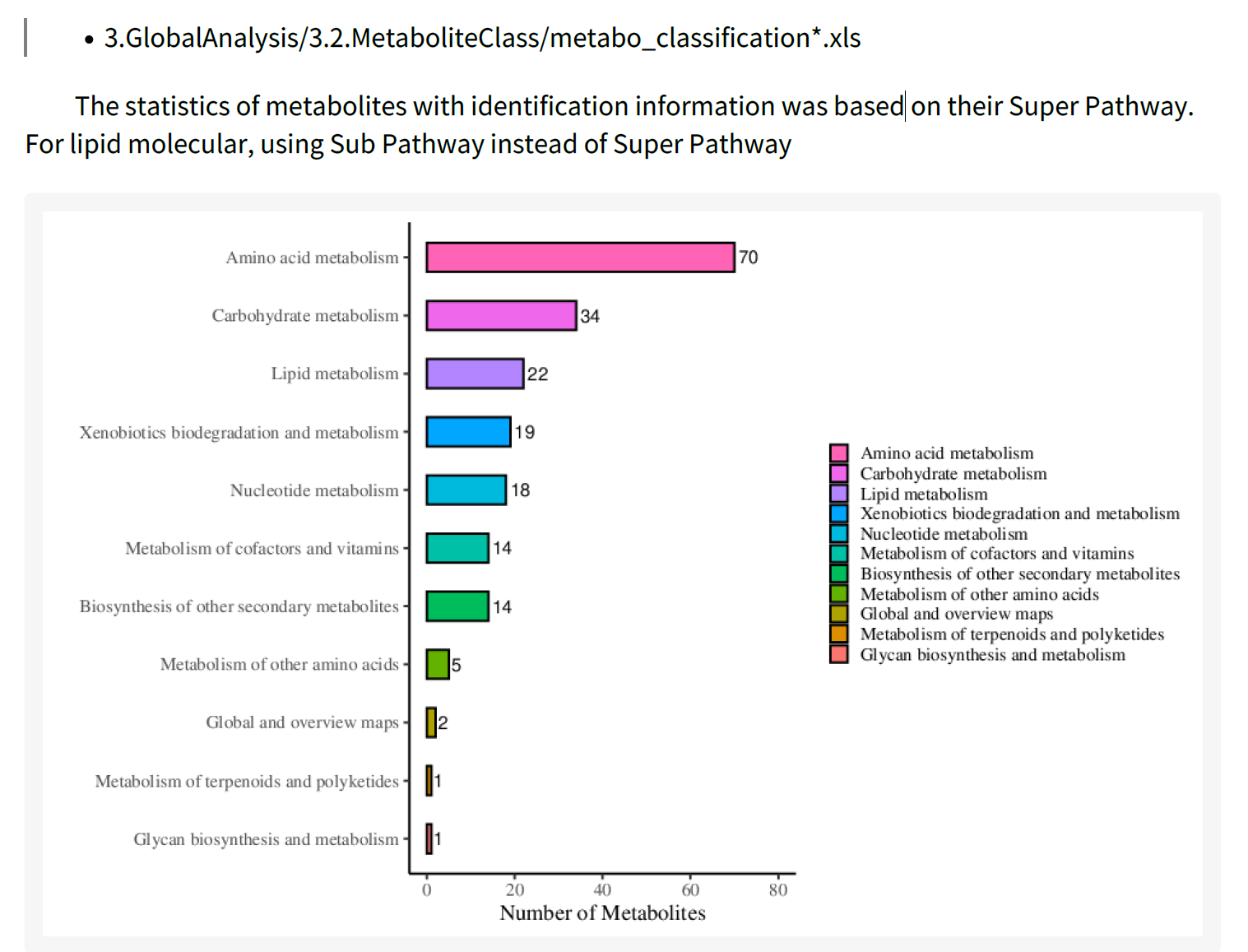
 Supplementary Figure 2.** Classification of all detected 432 metabolites with absolute quantification


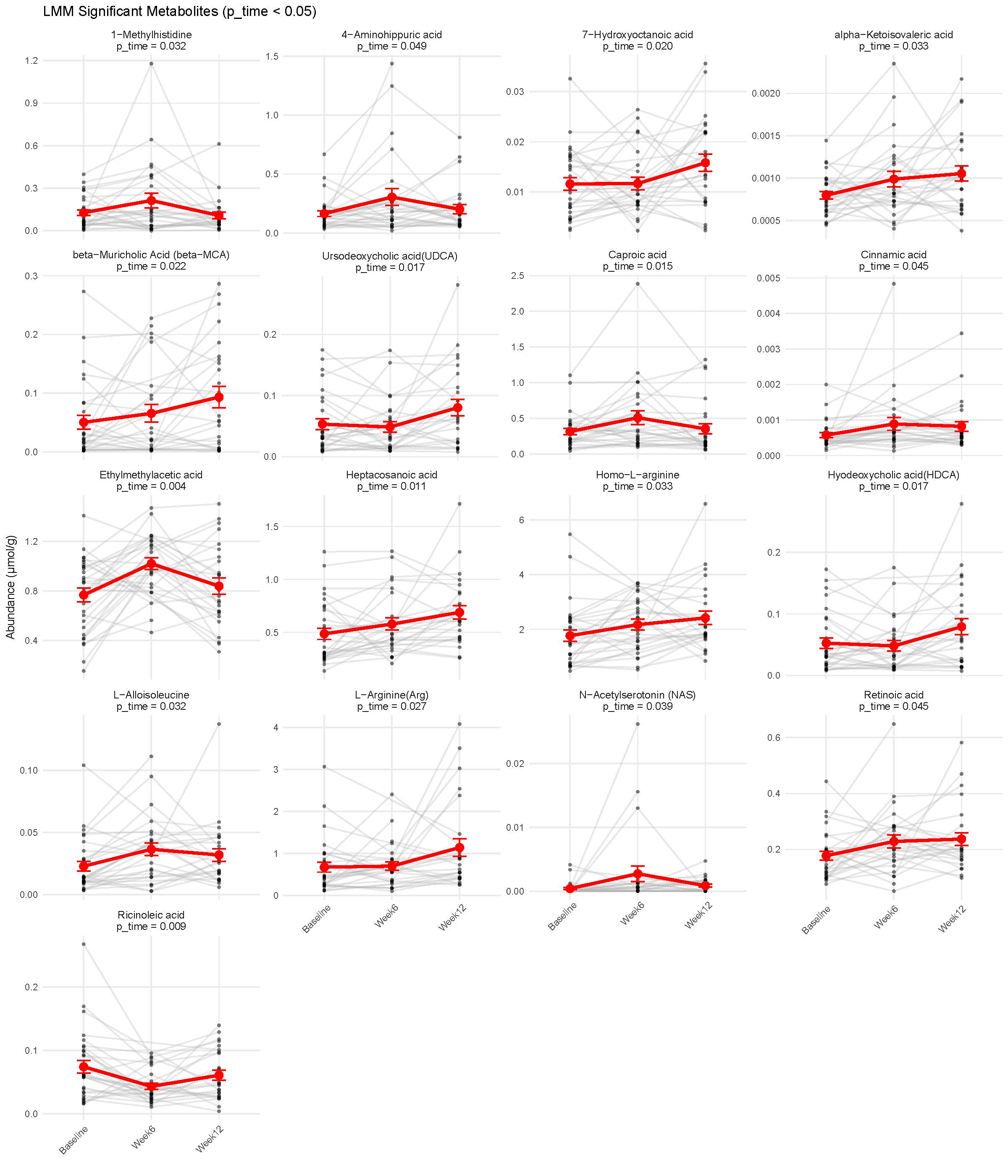
**Supplementary Figure 3**. Fecal metabolites that showed nominal changes during SCM06 treatment (p < 0.05, linear mixed models).


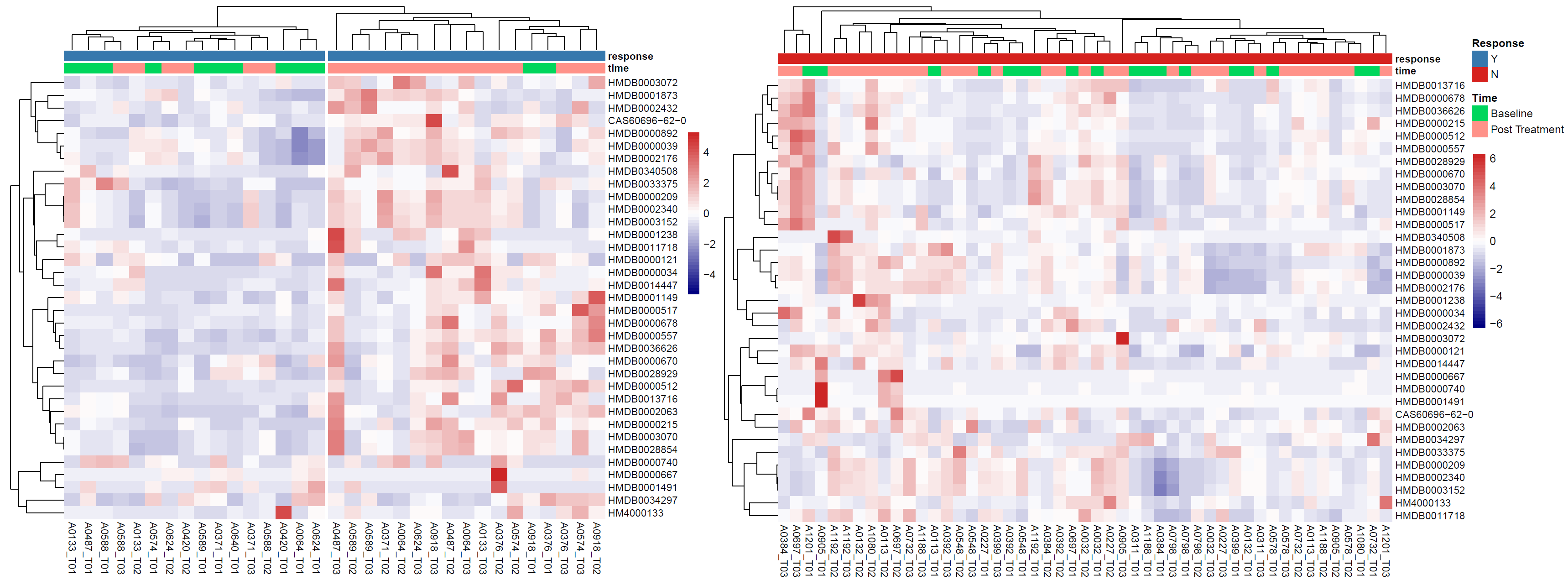


b


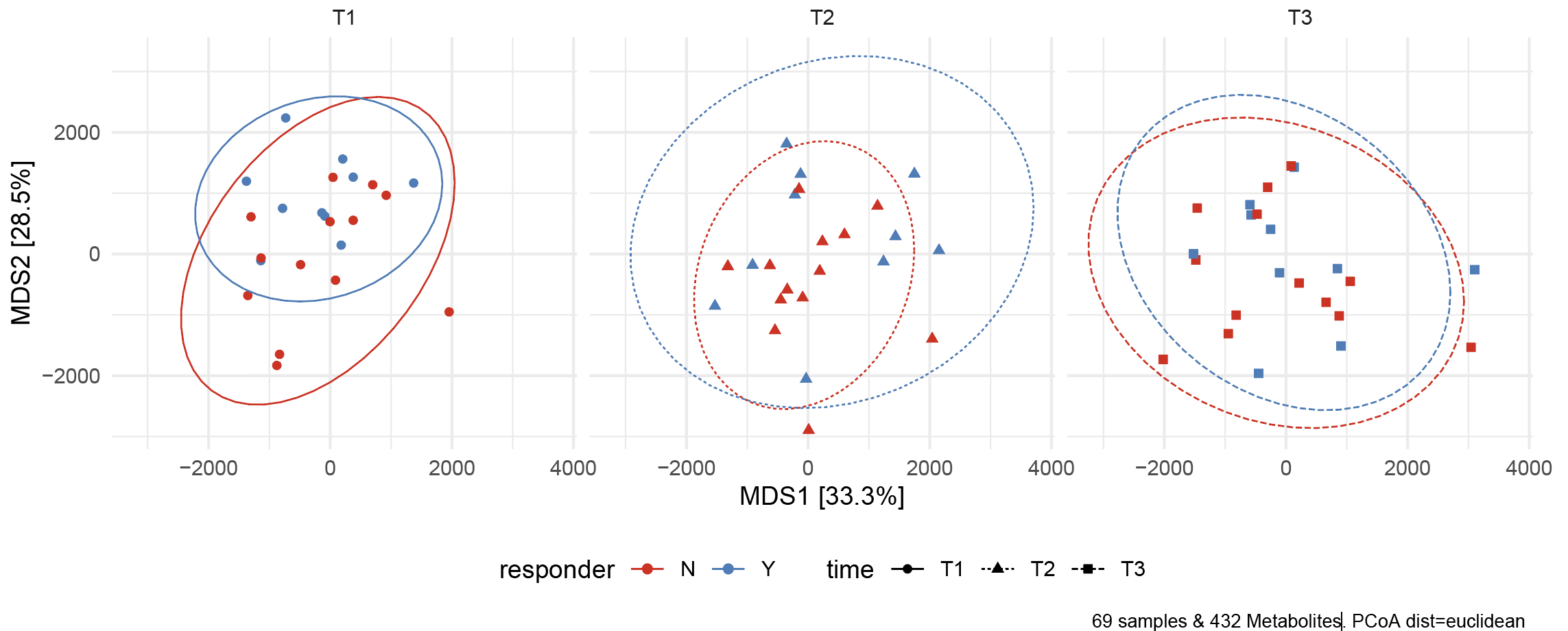


a

**Supplementary Figure 4.** a) Heatmap comparing the abundance of fecal metabolites between responders and non-responders. Only metabolites that exhibited significant changes following SCM06 intervention were shown. b) Overall fecal metabolome shifts in responders and non-responders.


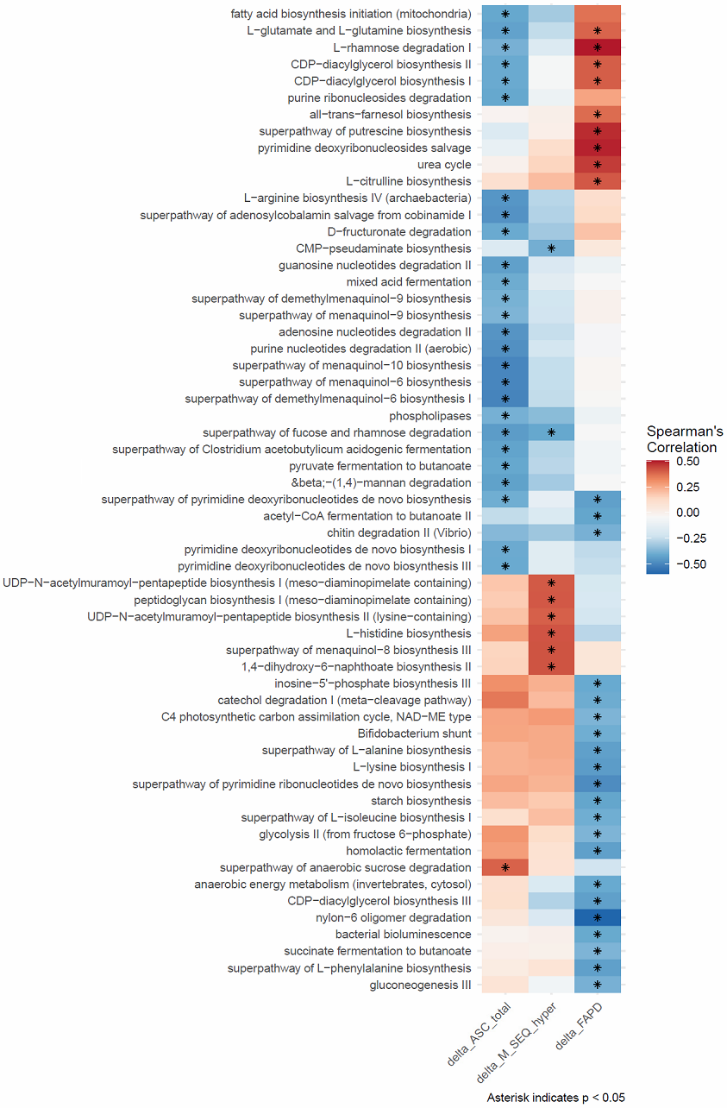


b

a


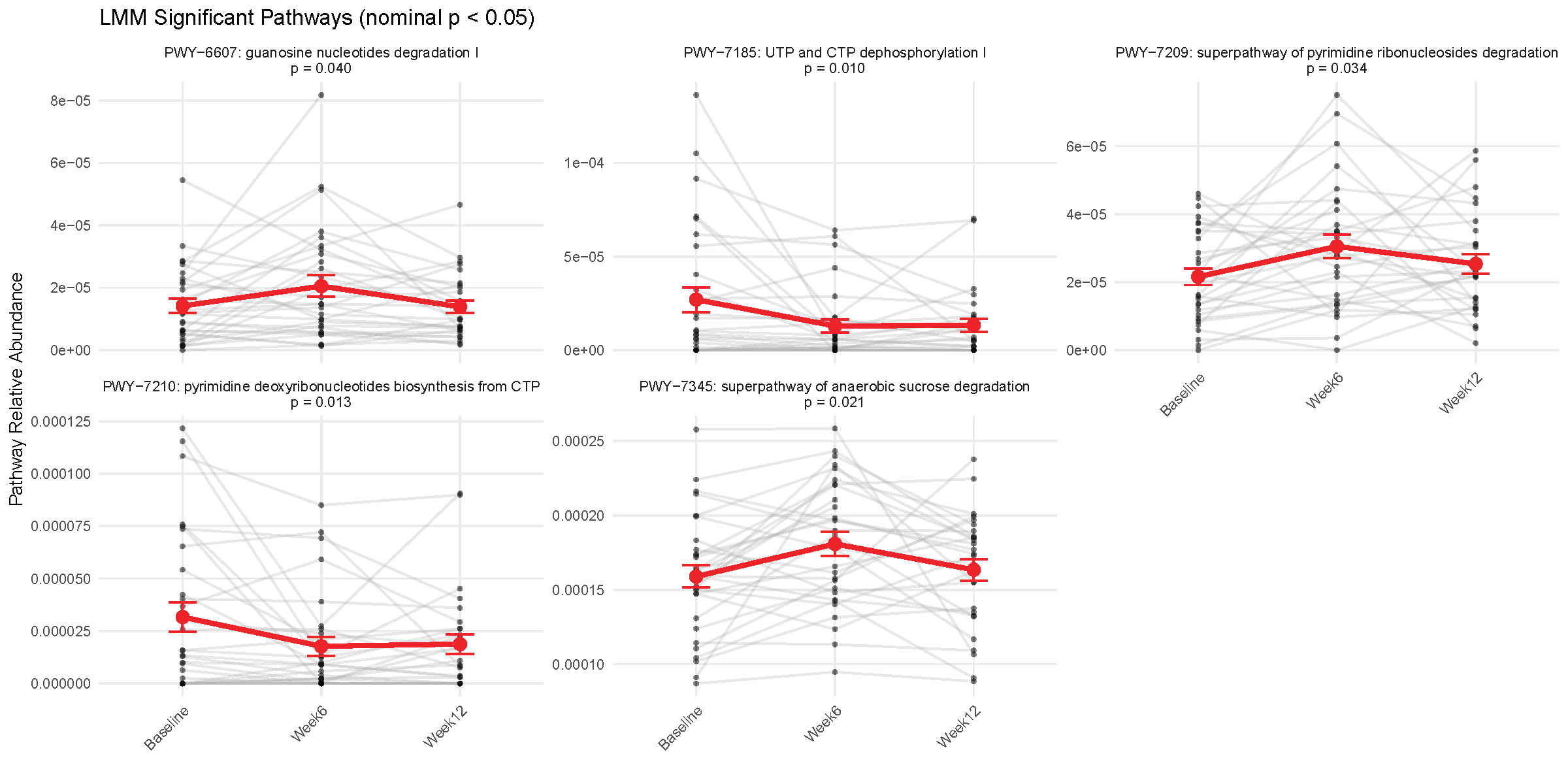


**Supplementary Figure 5.** a) Metabolic Functional Pathways that showed nominal changes during SCM06 treatment (p < 0.05, linear mixed models) b) Metabolic Functional Pathways that correlated with changes in anxiety (delta_ASC_total), sensory hyperresponsiveness (delta_M_SEQ_)hyper) symptoms, and functional abdominal pain disorders (delta_FAPD).


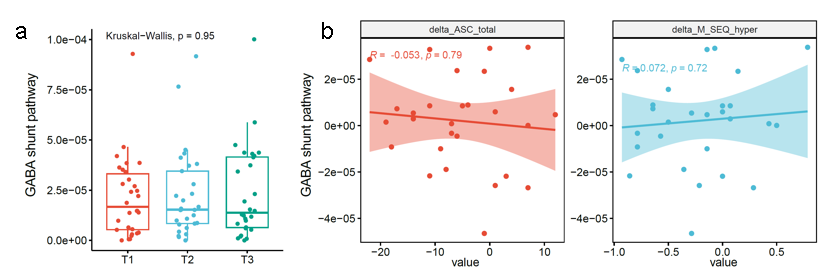


**Supplementary Figure 6.** a) Changes in the GABA Shunt pathway throughout the 12-week course of SCM06. b) Correlation of the GABA Shunt pathway and changes in anxiety (delta_ASC_total) and sensory hyperresponsiveness (delta_M_SEQ_hyper) symptoms.
